# Role of mannose-binding lectin on pneumococcal infections

**DOI:** 10.1186/cc10632

**Published:** 2012-03-20

**Authors:** J Solé Violán, I García-Laorden, F Rodríguez de Castro, A Payeras, J Ferrer Agüero, M Briones, L Borderías, J Aspa, J Blanquer, O Rajas, M García-Bello, J Noda, J Rello, C Rodríguez Gallego

**Affiliations:** 1 Hospital Dr Negrín, Las Palmas de Gran Canaria, Spain; 2 Hospital Son Llatzer, Palma de Mallorca, Spain; 3 Hospital Clínico y Universitario, Valencia, Spain; 4 Hospital San Jorge, Huesca, Spain; 5 Hospital de La Princesa, Madrid, Spain; 6 Hospital Universitario Vall d'Hebró, Barcelona, Spain

## Introduction

The role of mannose-binding lectin (MBL) deficiency (MBL2 XA/O + O/O genotypes) in host defences remains controversial. The surfactant proteins (SP)-A1, SP-A2 and SP-D, and other collectins whose genes are located near MBL2, are part of the first-line lung defence against infection. We analyzed the role of MBL on susceptibility to pneumococcal infection and the existence of linkage disequilibrium (LD) among the four genes.

## Methods

We studied 348 patients with pneumococcal community-acquired pneumonia (P-CAP) and 1,591 controls. A meta-analysis of MBL2 genotypes in susceptibility to P-CAP and to invasive pneumococcal disease (IPD) was also performed. The extent of LD of MBL2 with SFTPA1, SFTPA2 and SFTPD was analyzed.

## Results

MBL2 genotypes did not associate with either P-CAP or bacteraemic P-CAP in the case-control study. The MBL-deficient O/O genotype was significantly associated with higher risk of IPD in a meta-analysis, whereas the other MBL-deficient genotype (XA/O) showed a trend towards a protective role. We evidenced the existence of LD between MBL2 and SPs genes.

## Conclusion

The data do not support a role of MBL deficiency on susceptibility to P-CAP or to IPD. LD among MBL2 and SP genes must be considered in studies on the role of MBL in infectious diseases.

